# Mosquito Control Priorities in Florida—Survey Results from Florida Mosquito Control Districts

**DOI:** 10.3390/pathogens10080947

**Published:** 2021-07-28

**Authors:** Rishi Kondapaneni, Ashley N. Malcolm, Brian M. Vazquez, Eric Zeng, Tse-Yu Chen, Kyle J. Kosinski, Ana L. Romero-Weaver, Bryan V. Giordano, Benjamin Allen, Michael T. Riles, Daniel Killingsworth, Lindsay P. Campbell, Eric P. Caragata, Yoosook Lee

**Affiliations:** 1 Florida Medical Entomology Laboratory, Department of Entomology and Nematology, Institute of Food and Agricultural Sciences, University of Florida, Vero Beach, FL 32962, USA; kondapanenir@ufl.edu (R.K.); a.malcolm1@ufl.edu (A.N.M.); vazquezb@ufl.edu (B.M.V.); eric.zeng@ufl.edu (E.Z.); papilioninae@ufl.edu (T.-Y.C.); kyle.kosinski@ufl.edu (K.J.K.); aromeroweaver@ufl.edu (A.L.R.-W.); b.giordano@ufl.edu (B.V.G.); lcampbell2@ufl.edu (L.P.C.); e.caragata@ufl.edu (E.P.C.); 2 Mosquito Control Division, City of Jacksonville, Jacksonville, FL 32202, USA; benjamina@coj.net; 3 Beach Mosquito Control District, Panama City Beach, FL 32413, USA; michael@pcbeachmosquito.com; 4 Environmental Security Pest Control, Cantonment, FL 32533, USA; dan.killingsworth@pest.com

**Keywords:** mosquito control, vectors, Florida, survey, vector control, control priority

## Abstract

Florida lies within a subtropical region where the climate allows diverse mosquito species including invasive species to thrive year-round. As of 2021, there are currently 66 state-approved Florida Mosquito Control Districts, which are major stakeholders for Florida public universities engaged in mosquito research. Florida is one of the few states with extensive organized mosquito control programs. The Florida State Government and Florida Mosquito Control Districts have long histories of collaboration with research institutions. During fall 2020, we carried out a survey to collect baseline data on the current control priorities from Florida Mosquito Control Districts relating to (1) priority control species, (2) common adult and larval control methods, and (3) major research questions to address that will improve their control and surveillance programs. The survey data showed that a total of 17 distinct mosquito species were considered to be priority control targets, with many of these species being understudied. The most common control approaches included truck-mounted ultra-low-volume adulticiding and biopesticide-based larviciding. The districts held interest in diverse research questions, with many prioritizing studies on basic science questions to help develop evidence-based control strategies. Our data highlight the fact that mosquito control approaches and priorities differ greatly between districts and provide an important point of comparison for other regions investing in mosquito control, particularly those with similar ecological settings, and great diversity of potential mosquito vectors, such as in Florida. Our findings highlight a need for greater alignment of research priorities between mosquito control and mosquito research. In particular, we note a need to prioritize filling knowledge gaps relating to understudied mosquito species that have been implicated in arbovirus transmission.

## 1. Introduction

Currently, there are at least 84 mosquito species in the state of Florida [1,2]. The state lies within a subtropical region where the climate allows different mosquito species to thrive in specific regions and during specific seasons. For example, previous research on *Culex* mosquitoes showed *Culex nigripalpus* (Theobald) in a South Florida wastewater lagoon were most abundant during summer and fall, while *Culex salinarius* (Coquillett) and *Culex quinquefasciatus* (Say) were more common in winter or early spring [3]. Many of the mosquito species that become most active around summer, such as *Aedes aegypti* (Linnaeus), *Aedes albopictus* (Skuse)**, and *Cx. quinquefasciatus*, are prominent vectors of viruses that cause diseases such as Chikungunya, dengue, Zika, and West Nile, which cause millions of infections in humans around the world [4]. Other pathogens such as Eastern equine encephalitis virus (EEEV) [5,6] and St. Louis encephalitis virus [7,8] also occur in Florida.

Mosquito species vary in their distributions according to abiotic climate and biotic habitat factors, such as temperature, precipitation, and habitat competition, amongst others, with many of these factors differing substantially across the state. Habitat ecology (i.e., urban, suburban, rural, forested) also helps shape species population distributions due to the availability of preferred blood meal hosts, and areas with higher pathogen/host concentrations have a higher risk of containing mosquito vector populations [9]. Additionally, landscape also plays a role in species distributions by providing different types of larval sites [9].

Florida is also a prime target for invasive species. Factors such as a subtropical climate, extensive tourism, and a role as a hub for international commerce, have often led to invasive species being introduced into the Florida ecosystem [10,11]. Over the last two decades, prominent invasive species have included *Aedes japonicus* (Theobald)**, *Aedes scapularis* (Rondani)**, *Aedeomyia squamipennis* (Lynch Arribálzaga)**, *Culex coronator* (Dyar and Knab), and *Culex panocossa* (Dyar and Knab) [12,13,14,15,16,17]. This problem is not unique to Florida. For example, *Aedes pertinax* (Grabham) has been introduced in neighboring Caribbean [18] and *Culex declarator* (Dyar and Knab) and *Culex interrogator* (Dyar and Knab) have been introduced in south and central America [19,20]. Critically, many of these invasive species are also vectors, and their presence in Florida could help novel pathogens become established or lead to more widespread transmission of pathogens that have historically been observed in the state [17,21,22]. Depending on the blood meal host preferences of these species, there may also be increased risk that mosquito-transmitted pathogens that circulate amongst animals might cross over and infect humans.

Historically, diseases caused by mosquito-transmitted pathogens have been a major issue in Florida. In the early 1900s, when the link between mosquitos and disease was revealed, Dr. Joseph Y. Porter and other officials formed the Florida Anti-Mosquito Association [23]. Soon thereafter, communities and counties were authorized to create Florida Mosquito Control Districts (FMCDs) to defend themselves against disease-causing and pestiferous mosquito populations. As of 2021, there are 66 state-approved FMCDs, of which 11 are special taxing districts governed by elected officials outside the governance of county or city officials and are mandated by the State of Florida legislature. Early approaches to mosquito control included dynamite ditching, diking, and dewatering. By the mid-1940s, the use of aerosolized chemical insecticides such as dichloro-diphenyl-trichloroethane (DDT) and temephos became increasingly common methods of mosquito control [24]. However, it became apparent that chemical control alone was not enough to mitigate mosquito populations, and growing concerns of insecticide resistance, toxicity, nontarget effects, and environmental persistence resulted in calls for innovation and new technologies. FMCDs now practice diverse forms of larviciding and adulticiding, but also conduct surveillance of mosquito populations, and test the efficacy of mosquitocidal formulations. Some districts also run educational outreach programs that teach students and the general public about mosquito control, biology, and surveillance. Control priorities differ between districts depending on the mosquito species and pathogens in the area. 

To help maintain effective control of mosquito populations, FMCDs often work in partnership with research facilities. These collaborations can provide opportunities for the provision of training on new control approaches, surveillance and analysis techniques, or mosquito identification, as well as access to literature or knowledge to help FMCDs select safe and effective control approaches, assistance with analyzing primary data generated by FMCDs, and also access to diverse funding opportunities. Research facilities benefit through improved access to field sites, and the ability to conduct higher quality and more impactful research programs targeted to directly address the needs of a key stakeholder group. Historically, these collaborations have proven fruitful, and have resulted in major advances in control techniques, such as the production and evaluation of new insecticide agents, including methionine [25], and the evaluation of novel deployment methodologies and barrier treatments [26]. They have also provided vital insight into the distributions of key pathogens, vector and host species, and the role of environmental changes in mosquito biology [27].

In a landscape containing many diverse mosquito species and pathogens, maintaining effective mosquito control has direct consequences to public health. FMCDs must also deal with added complexity in the form of invasive mosquitoes, outbreaks of diseases caused by mosquito-transmitted pathogens, and the potential for insecticide resistance to arise amongst the mosquito populations they target, which might necessitate changing to a different control approach. As such, there are wide range of control priorities across the FMCDs. There is consequently a need for mosquito researchers to stay aware of FMCD activities and priorities in order to provide effective support. Accordingly, we conducted an initial survey, which will form part of a long-term stakeholder engagement effort intended to improve understanding of current FMCD control priorities, common control practices, and also highlight the mosquito control perspective on future mosquito control research needs. 

## 2. Results

### 2.1. FMCD Priority Species for Mosquito Control

**Figure 1:**
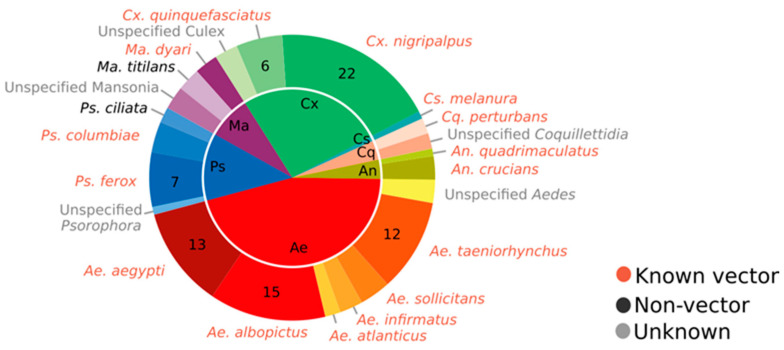
Mosquito species or genera mentioned as being top control priorities by FMCDs. As part of the survey, each of the 34 responding FMCDs mentioned up to 3 unranked mosquito species that were their top control priorities. Survey data revealed the responding FMCDs considered 17 distinct species to be control priorities. This chart displays the percentage of total mentions for each mosquito species (inner circle) or genus (outer circle). Common colors represent species from the same genus. Numbers in the pie chart describe the number of FMCDs that mentioned that specific species as being a control priority. Red text indicates species known to vector pathogens of human diseases, black text indicates a non-vector, and gray text indicates that vector status is uncertain.

We received 34 total responses from the 66 FMCDs that we contacted. Each district mentioned up to three species as priority control targets, with these species being unranked (Figure 1). Across these districts, a total of 17 distinct species were named as priority control targets. The responses of some FMCDs indicated targets only at the genus level. Overall, the data showed that the most often mentioned species was *Cx. nigripalpus*, which was a control priority for 21 out of 34 responding FMCDs (62%), while the second most mentioned was *Ae. albopictus*, a priority for 15/34 FMCDs (44%).

Assessing the survey data at the genus level, we saw that there were 52 mentions for the genus *Aedes*, but these mentions came from only 44% of the FMCDs that responded. A total of seven different *Aedes* species were mentioned as priorities, with the most frequently mentioned being *Ae. albopictus* at 44% of FMDCs, *Ae. aegypti* at 38%, and *Ae. taeniorhynchus* (Wiedemann) at 35%. Genus *Culex* was mentioned by 79% of the FMCDs that responded, but only two specific species were mentioned, *Cx. nigripalpus* and *Cx. quinquefasciatus*, with the former a control priority for 65% of FMCDs and the latter for 18%. Other frequently mentioned species included *Psorophora ferox* (von Humboldt), which was identified as a major control priority by seven FMCDs. Of the 17 species mentioned, 70% were vectors of known pathogens, while the remaining 30% were nuisance biters that have not been implicated in pathogen transmission.

**Figure 2:**
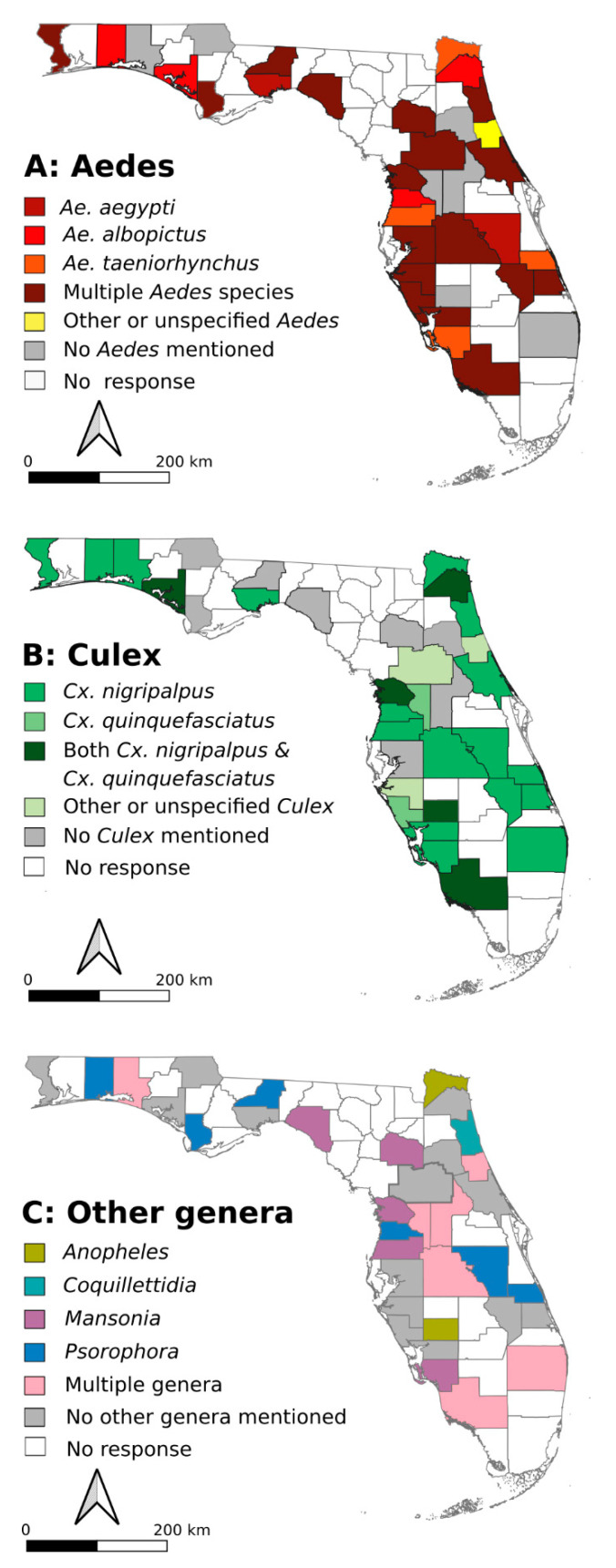
Maps outlining priority species for mosquito control in Florida, at the county level. These maps highlight priority mosquito species for control in different regions of Florida for genus *Aedes* (**A**), genus *Culex* (**B**), and other genera of mosquitoes (**C**), and demonstrate that many neighboring counties had similar priorities.

When comparing the results with the geographic location of the FMCDs, we saw that 19–21 counties had similar control priorities to their neighbors, especially with respect to mosquitoes from the genera *Aedes* and *Culex.* Outside of those two genera, there were few clear trends that suggested that there were region-specific priority species. FMCDs located in counties in central Florida commonly reported *Aedes* species as their top control priority (Figure 2A). Most of the counties that prioritized control of *Aedes* mosquitoes were concerned about multiple *Aedes* species. Interest in controlling *Cx. nigripalpus* was widespread geographically, likely reflecting the fact that it was the most common control priority in the dataset (Figure 2B). Four out of the five FMCDs which reported a need to control multiple *Culex* species were located on the Gulf Coast (Figure 2B). Some southern FMCDs such as Collier and Palm Beach Mosquito Control Districts (MCDs), as well as multiple MCDs in the middle of Florida (Lake, Sumter, and Polk), reported multiple genera as their control priority. FMCDs that reported *Mansonia* as their control priority were mostly on the Gulf Coast (Figure 2C). *Psorophora* were only reported as a control priority in Central Florida and the Panhandle (latitude 27.5 or above; Figure 2C). Of all of the genera mentioned, *Anopheles* or *Coquillettidia* were least mentioned as top control priorities. 

### 2.2. Activity Times of FMCD Priority Control Species

**Figure 3:**
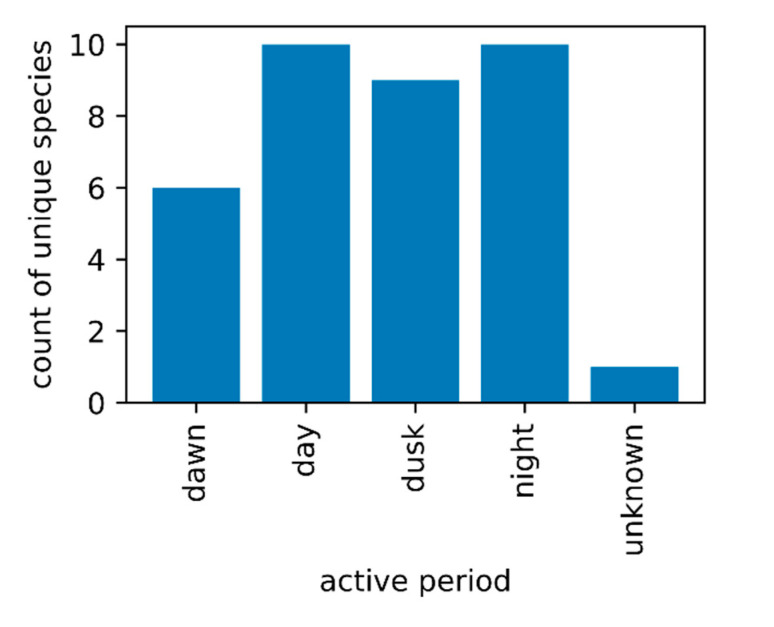
Daily peak activity period of priority species for mosquito control in Florida. This figure shows counts of mosquito species distributed across their daily activity periods, based on a literature review. Several species were recorded as being active across multiple time periods. The data indicated that FMCDs were interested in targeting mosquitoes with diverse activity periods, with the majority of these species approximately evenly split between day, dusk, and night host-seeking and biting behaviors.

Through a literature review, we assessed the activity times for the 17 mosquito species identified by FMCDs as being control priorities (Figure 3). A single species was added in multiple active period categories if it had a broader spectrum of activity time. A total of 15 mosquito species were identified as being crepuscular, active primarily at dawn (6 species) or dusk (9 species), while 10 species were recorded as being diurnal and a further 10 as being nocturnal. For one species, *Mansonia dyari* (Belkin, Heinemann, and Page), information on activity was not obtained.

### 2.3. Common Mosquito Control Methods Used by FMCDs

**Figure 4:**
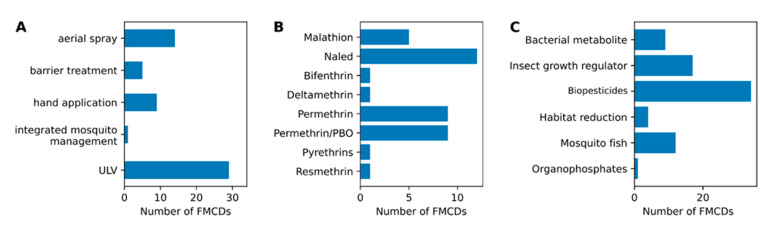
(**A**): Common adult control methods used in Florida. (**B**): common insecticides used for mosquito control in Florida. (**C**): common larval control methods used in Florida. Our survey data indicated that FMCDs utilized a range of approaches to control mosquito populations in their districts. The most common format of adulticide deployment was through the use of ULV, with 100% of FMCDs reporting the use of biopesticides of microbial origin as their typical choice for larval control. Commonly used biopesticide products included *Bacillus thuringiensis* subspecies *israelensis* (Bti), a biological or a naturally occurring bacterium found in soils, and *Lysinibacillus* *sphaericus* (commonly referred to as Bs), a common soil inhabiting bacterium. The second most commonly used format (50% of FMCDs) was larviciding using the juvenile growth inhibitor, methoprene, which prevents normal molting. Other methods of larval control involve the use of mosquito fish or bacterial metabolites, such as *Saccharopolyspora spinosa* chemical derivatives.

The survey response data indicated that most FMCDs were utilizing multiple approaches to control adult mosquito populations. Spraying adulticidal chemicals was the most common intervention format, and the most common method of deployment involved the use of truck-mounted ultra-low volume (ULV) adulticide application (Figure 4A). This was utilized by 100% of responding FMCDs. Aerial spraying was the second most commonly used method of deployment, with this being practiced by 48% of the responding FMCDs. The adulticidal active ingredients used by FMCDs fell into one of two classes available for use: organophosphates or pyrethroids. Naled and malathion were the most commonly employed organophosphates, and were used by 12 and 5 FMCDs, respectively (Figure 4B). The pyrethroid class accounted for the remaining 22. Permethrin or permethrin/PBO were the most commonly employed pyrethroids and were used by nine FMCDs. 

### 2.4. Research Questions That Florida Mosquito Control Districts Identified to Be Important

**Figure 5:**
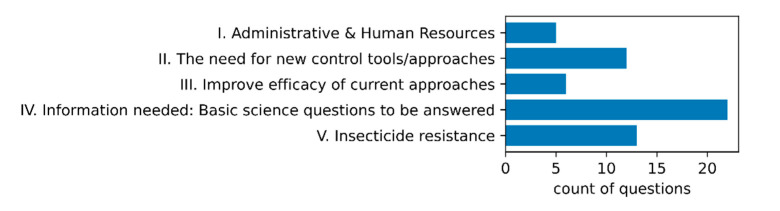
FMCD research priorities. Each FMCD was asked about research questions that they would like to see answered in order to improve their mosquito control programs. These questions could be grouped into five categories (I–V), with the most common type of question falling into the category of basic science questions that focused on addressing knowledge gaps linked with mosquito biology, and vector and pathogen surveillance.

We asked each FMCD about research questions that needed to be addressed in order to help improve their control efforts. The answers we received suggested that FMCD research priorities fell into five broad categories (Figure 5). The most common FMCD research priorities were focused on questions of basic science, including gathering evidence and information, predominantly about vector populations in order to inform surveillance and control decisions. Key topics in this category included improving understanding of vector dispersal, interspecies interaction, non-target effects, efficacy and residual effects of control products and their environmental impact, spatial modeling and risk prediction, biology of neglected species, better outreach/citizen-assisted surveillance tools, and increasing capacity to be proactive in combating mosquito-borne diseases (Table S1). Another key research priority was insecticide resistance, with more than 20 FMCDs interested in the mechanisms and prevalence of insecticide resistance, and approaches for managing resistant mosquito populations. In line with this issue, a further 18 questions were concerned with improving existing approaches to control (6 questions) or the need to identify novel tools for use in control and/or surveillance (12 questions; Figure 5). The final category concerned issues related to human resources and operational efficiency and included topics such as the need for improved employee training. The full questions are provided in Table S1. 

## 3. Discussion

“What is the most important mosquito species in Florida?” It’s a deceptively simple question, but the answer is complicated by numerous factors. Some mosquito species are not abundant but are important vectors of pathogens that cause human or veterinary disease; while some are abundant, but merely a public nuisance. Mosquito species occupy different environmental niches, which not only affects their local abundance, but also the strategy required to control their populations. In line with this complexity, our results indicated that each FMCD attempts to utilize its resources and shapes its control program to survey and control diverse targeted species. As such, our data suggest that there are multiple, diverse approaches taken to mosquito control in Florida, with control priorities and approaches varying amongst FMCDs (Figure 2 and Figure 4). One notable finding amongst our data was that FMCDs are concerned not only with major vectors such as *Ae. aegypti* and *Ae. albopictus*, but also with understudied vector and nuisance biting species. It is important to consider how the decision to prioritize these species aligns with information on their status as vectors and their historical links to outbreaks in the state. 

### 3.1. Aedes albopictus and Aedes aegypti

The yellow fever (*Ae. aegypti*) and Asian tiger *(Ae. albopictus*) mosquitoes are efficient vectors for dengue, Chikungunya, Zika, and yellow fever viruses, and are common in many parts of Florida [28]. Consequently, it was not surprising to find that they were named as important control priorities for many FMCDs. Both are invasive species in Florida, with the former having been introduced during the colonization of North America, and the latter in 1985 [29,30,31]. Although they originated on separate continents and have their own evolutionary history, they are behaviorally similar: they are aggressive diurnal biters and prefer to lay eggs in vessels common in urban areas such flowerpots or plastic containers.

*Ae.aegypti* and *Ae. albopictus* are progressively expanding their geographical range in the continental US [32,33], and have recently been detected as far north as Wayne County, Michigan and southern Ontario, Canada [34]. They use hitchhiking strategies for long distance dispersal to colonize new places [35,36], demonstrating the need for active surveillance in multiple regions to identify introduced mosquitoes prior to population establishment in a location. 

Larval control for these species is challenging because they often live in small pockets of water collected in natural and manufactured containers such as ornamental bromeliads, pet food bowls, flowerpot saucers, water collected inside tires that are exposed to the environment, and various discarded garbage, leaving FMCDs with the impossible task of locating then treating or removing all potential larval habitats. Furthermore, adult control is complicated by high levels of insecticide resistance [37,38,39], and diurnal activity patterns that reduce the efficiency of adulticiding operations. Both adults and larvae are often associated with human housing to which it can be difficult for control personnel to gain access. For this reason, many Mosquito Control Districts throughout the US including Florida make significant efforts to educate the public in physical control (i.e., removal of standing water sources) so that they can be part of the active surveillance and control by removing potential breeding sources themselves. 

### 3.2. Culex nigripalpus

Perhaps the most interesting finding from our data was that *Cx. nigripalpus* was selected as a control priority across the greatest number of districts. This species has a high level of medical relevance and control priority in Florida. It is found in all 67 counties and is abundant year-round due to the prevalence of standing water sources, particularly after flooding and tropical storms, which are frequent in Florida during the rainy season (May through October). Female *Cx. nigripalpus* lay eggs in virtually all aquatic habitats, but research has shown that they prefer freshly flooded sites that stay saturated for 10–14 days after initial flooding [40]. They are opportunistic, nocturnal blood feeders, with hosts ranging from frogs to humans [40]. Critically, *Cx.* *nigripalpus* is the primary vector for St. Louis encephalitis virus (SLEV) [40], and a suspected vector for Eastern equine encephalitis virus (EEEV) and West Nile virus (WNV) [41]. The last major SLEV outbreak in Florida took place in 1990, with 223 diagnosed cases. However, by extrapolating based on a ratio of asymptomatic to symptomatic cases of 300:1 [42], a more accurate estimate of case numbers during this outbreak was ~66,900. Combined with this species’ rapid oviposition, breeding, and opportunistic blood-feeding behaviors, *Cx. nigripalpus* is a formidable vector species and serious threat to human and veterinary health in Florida.

The microbiota of mosquito species can be a factor that can also influence disease transmission for both virus [43,44,45] and parasitic pathogens [45,46]. The sampling of female *Cx. nigripalpus* from Vero Beach, Palmetto Inland, and Palmetto Coast showed significant differences in their microbiota communities [47]. Characterizing these population-specific differences in bacteria communities may help with identifying potential symbionts that may lead to new mosquito control methods [47]. More research needs to be conducted to see if there is any connection between microbiota variations and disease transmission efficacy. Studies of vector competence for this species remain a major challenge due to difficulties maintaining colonies under laboratory conditions [48].

### 3.3. Aedes taeniorhynchus 

*Aedes taeniorhynchus* was identified as one of the top three priority *Aedes* species by the Mosquito Control Districts in Florida. They have a broad distribution across salt marshes in North America, Central America, South America, the Caribbean islands, and are found along the entirety of the Florida coast [49]. They are identifiable by characteristic black scaled femur and tibia and the absence of the "spots" or "medial longitudinal pale stripe" on the abdominal terga [50]. Eggs are laid in moist soil above the waterline. Once the soil is flooded with water, it triggers hatching. In Florida, *Ae. taeniorhynchus* populations develop predominantly in the grass marshes of the North and Panhandle regions, the east central coast scrub salt marshes, and the mangrove swamps of South Florida [51]. After hatching, the larval stage takes about 5–15 days to complete. The larvae can survive in water with very high salinity levels [49]. Adults can often be found quite far inland due to their long flight range [52]. This species occurs across the whole of Florida due to their flight range, not because they are completing their full life cycle in all Florida counties.

*Aedes taeniorhynchus* serves as a bridge vector, and is known to feed on birds, mammals, and reptiles. This species has medical relevance since it is a known vector of EEEV and Venezuelan equine encephalitis virus (VEEV) [53,54] and a likely vector of WNV [55,56]. 

### 3.4. Mosquito Control Priority Versus Research Interest

**Figure 6:**
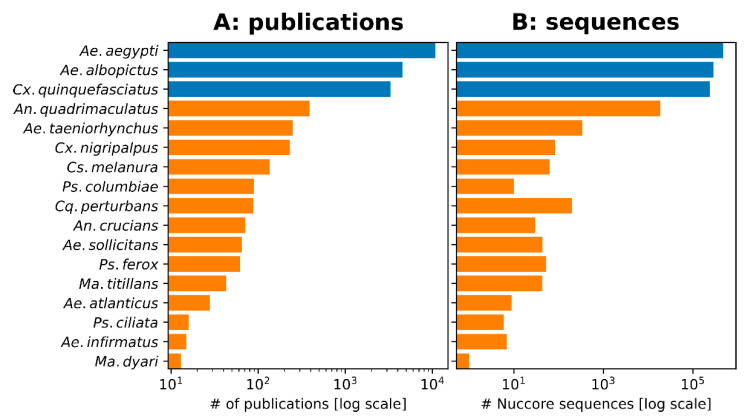
Alignment between FMCD priority control species and the current state of research on those species. Number of publications in PubMed (**A**) and published sequences from NCBI Nucleotide database (**B**) for each species identified to be an important control priority for FMCDs (data as of May 2021). The major vectors are marked in blue bars and the ‘understudied’ species are marked in orange bars. We classified a species as ‘understudied’ if the number of peer-reviewed publications on PubMed was 10-fold lower, or less, than the median publications for the three major vectors.

To compare the relative interest in mosquito species identified as control priorities by FMCDs with the scientific literature, we conducted searches on each species in the NCBI PubMed database (https://pubmed.ncbi.nlm.nih.gov) (30 May 2021) (Figure 6A). This search revealed that each of the three vectors with prominent global distributions, *Ae. aegypti*, *Ae. albopictus*, and *Cx. quinquefasciatus*, had each been published about thousands of times. The remaining 14 FMCD priority control species were described in, at most, several hundred papers, although for some species this number was far lower. An additional analysis of the NCBI Nucleotide database (https://www.ncbi.nlm.nih.gov/nucleotide/) (30 May 2021) revealed a similar trend in terms of genetic resources, as for 13/17 mosquito species, there were fewer than 1000 DNA or RNA sequences in the database (Figure 6B).

Based on the results of our survey, we have detected a major misalignment of priorities between FMCDs and scientific resources devoted to the studies of certain mosquito species. This is most notable for *Cx. nigripalpus* and *Ae. taeniorhynchus*, which, as mentioned above, are major priority species for many FMCDs and under described in the literature; however, this disparity also exists for the other 12 species identified in the survey. The limited mention of *Cs. melanura* by FMCDs as a control priority is also noteworthy given that (1) Florida has human and horse cases annually, (2) Florida was listed as one of the states where most cases came from between 2010 and 2019 [57], and (3) the species is relatively high in publication globally amongst understudied species (Figure 6). This is problematic as successfully developing and implementing a mosquito control strategy goes hand in hand with possessing rigorous and accurate information on the likely distribution of a mosquito species, its host preferences, and when and where they are likely to be host seeking. These factors can vary greatly between mosquito species, or even within species, depending on their location and/or genetic background [58,59]. Without sufficient, high quality information on specific mosquito targets, developing persistent and effective control programs may prove challenging, particularly during outbreaks. With this in mind, there is a clear need to conduct more research on understudied species, such as those we have described above.

### 3.5. Active Time of Adult Mosquitoes

Knowledge of when specific mosquito species are actively host seeking is critical to the success of any intervention, but also its broader impact on the treated area. From a public health perspective, this knowledge can allow residents to engage in self-protecting behaviors when enjoying outdoor activities. Properly timing adulticidal interventions is an important step in reducing or eliminating the impact of these interventions on non-target insects, including pollinators, such as *Apis mellifera* [60]. The timing of wide area ultra-low volume spatial applications of aerosolized pesticides must take into account the behavior of each species’ peak host-seeking activity time or the insecticide droplets will not come into contact with the mosquitoes [61]. In Florida, most ultra-low volume spatial applications occur at dusk to target crepuscular species [24]. However, not all vector and nuisance species are crepuscular. For instance, *Ae. aegypti* and *Ae. albopictus* are considered to be diurnal host-seeking species [62], while many species, including *An. quadrimaculatus* are nocturnal biters. From a mosquito control perspective, it is important to leverage species-specific knowledge of activity periods to give any intervention the greatest chance of impacting the target species [63]. Our data revealed that information on activity periods was available for 16/17 FMCDs priority species, all except *Ma. dyari*. It is important that these data be made broadly available, and also considered when developing control approaches that target multiple species with distinct activity periods.

### 3.6. Adulticide Choices

FMCDs conduct adulticiding procedures when deemed necessary to control mosquito population densities. Our data indicated that organophosphates were a common adulticide choice in the state of Florida. Naled and malathion are the two most utilized organophosphates by FMCDs. A third organophosphate, chlorpyrifos, has been removed from use [24]. Malathion is considered “slightly hazardous”, while naled is a corrosive agent and “extremely hazardous”. Although organophosphates have an extremely high efficacy, adulticiding using organophosphates is only performed after extensive consideration and contemplation by FMCDs due to their toxicity [24]. As such, their implementation is limited to reduce human exposure.

Adulticiding by using pyrethroids is becoming fairly common amongst FMCDs [38,39]. Pyrethroids offer a high degree of efficacy with characteristically lower environmental or human toxic side effects, providing an environmentally safer alternative to organophosphates. The use of piperonyl butoxide (PBO) has additionally increased the application frequency of the pyrethroids. PBO enhances the toxicity of the pyrethroid compound, even proving effective against resistant populations [64]. What was not captured in our survey was the fact that FMCDs often rotate between different adulticides, and will use organophosphates to control mosquito populations that are becoming more resistant to pyrethroids. 

### 3.7. Larval Control Choices

Our data highlighted that the most common choice for larvicides amongst responding FMCDs was the application of biopesticides of microbial origin, specifically Bti and Bs. These biopesticides are highly specific, and only affect insects from order Diptera (Bti) or certain species in the Culicidae family, leading to lower mortality rates amongst nontarget species than for other types of larvicides. Though effective, a dependency on Bti/Bs could potentially lead to the development of resistance in exposed populations, although this has not yet occurred in nature [65,66]. Integrating other control measures alongside larvicidal practices would be beneficial in maintaining current susceptibility level against these biopesticides. 

The second most popular FMCDs choice for larval population control is IGRs, with methoprene being used most abundantly. The mode of action for IGRs is to mimic juvenile hormones, altering or impeding the developmental cycle, resulting in adult sterility or deformities [67]. Methoprene is effective against a diverse range of mosquito genera with very low toxicity to mammals, invertebrates except lobsters [68], fish, and birds. In the environment, methoprene degrades rapidly having a 10-day half-life. Aquatic field studies, in which IGRs were directly placed into water sources, yield low residual concentrations, less than 0.5 µg/kg, in catch basins [69]. Lack of impact on the environment makes methoprene a popular control practice for federal and agricultural land. 

Use of *Gambusia*, or mosquitofish, is a biological control measure that several FMCDs use to target larval populations [70,71]. Mosquitofish are small freshwater carp that can be placed in drainage ditches, ponds, or any other bodies of water with adequate depth and connectivity. *Gambusia* can be collected from fish hatcheries, while colonies can easily be maintained on-site at individual FMCDs. Fortuitously, *Gambusia* are not affected by Bti or IGRs meaning that these methods can be utilized concurrently [71]. 

Another important control program practiced by FMCDs is source reduction, where potential larval habitats are targeted and eliminated. This can involve removing or emptying containers that house standing water, removal of garbage, turning over empty buckets or covering them with lids, and daily transfer of clean water into birdbaths. 

### 3.8. The Need for Coordination between Mosquito Surveillance, Control, and Research

Many FMCDs currently cooperate with one another, to varying degrees. This can include activities such as sharing research data, communicating information on disease or population outbreaks, or jointly evaluating new methods of insecticide dispersal [1]. As the results of our survey show, certain vector species, namely *Cx. nigripalpus*, *Ae. aegypti*, and *Ae. albopictus*, are clear control priorities for many FMCDs, including neighboring FMCDs. This alignment of priorities presents an opportunity for these FMCDs to expand their collaborations beyond sharing information and work together to counteract the threat posed by these species. While Florida might be an ecologically diverse state, the ecological factors associated with niches for specific mosquito species [72] are not restricted by geopolitical boundaries that define municipalities or the different FMCDs [2,17,73]. As such, specific mosquito populations might be expected to span district boundaries or migrate from district to district, especially in areas with high human population density [74]. This highlights the need for public health abatement districts, such as FMCDs, to communicate, collaborate, cooperate, and implement an integrated managed approach to surveillance and the suppression of mosquito pest populations, particularly if their specific goals are already well aligned [16,17,18]. 

When control priorities between neighboring districts are not aligned, which we also saw in our data, there is still a need to establish integrated approaches and clear channels of communications, particularly relating to issues of surveillance and sudden changes in species distributions, the appearance of invasive species, or the detection of pathogens [14]. If identified through one district’s surveillance program, effective communication of these data can help surrounding districts decide whether rapid shifts in their operational priorities are warranted. With 66 distinct districts and diverse control priorities there are clear benefits to be obtained by higher level coordination; perhaps through the Florida Mosquito Control Association Research Advisory Committee, the Florida Coordinating Council on Mosquito Control, or a working group that could facilitate communication between FMCDs with the goal of enhancing surveillance and control outcomes. This would serve as an ideal point of input for incorporating data and expertise from medical entomology research. 

### 3.9. Research Questions for Future Investigation

Mosquitoes are a clear threat to public health, with this threat having major economic implications. Yet with multiple potential vectors of many different pathogens often present in an area, it can be difficult for Mosquito Control Districts to decide where and how to devote their resources. Our survey data outlined the fact that FMCDs often had distinct priorities when it came to which mosquito species to target and how to target them (Figure 2 and Figure 4). Our survey also included a question about the research priorities of each district, and from the responses it was clear that the overwhelming majority of districts were interested in research that could help them optimize their control efforts. They were interested in information that could help them improve their current approaches, but also learning about novel approaches that they might turn to in the future. 

Closely related to this was an identified need to improve understanding of the biology and ecology of their priority species. Many districts identified a need to improve understanding of the basic biology of their mosquito targets, or to understand the insecticide resistance status of populations in their jurisdictions. For example, by increasing awareness of activity times to identify the optimal time to use pesticides or place traps, or understanding whether the pesticides they were using had an impact on the environment or non-target species. Research has demonstrated that human-induced changes to the environment can impact mosquito behavior and such changes should be considered as part of mosquito control decision making. For instance, light pollution has been shown to alter activity periods for certain mosquito species [75], meaning they might need to be targeted at different times of day than expected based on their previously described behavior. These examples highlight a need for researchers to address knowledge gaps that might prove critical to the success of particular control approaches, or to decision making relating to dispersal formats and timing, or the selection of alternative approaches. They also highlight the need for researchers to establish and maintain effective channels of communication with control districts and other stakeholders.

The idea of research and mosquito control interests operating in conjunction will likely prove invaluable into the foreseeable future, given that we are now seeing a changing control landscape. Over the past decade, several next generation mosquito control strategies have been tested in nature, with a view to supplementing or even replacing the types of interventions typically conducted by mosquito control districts. These new approaches include the use of the bacteria *Wolbachia*, which can be deployed at the city level to immunize mosquito populations against pathogens such as DENV [76]. Similarly, the Florida Keys Mosquito Control District has been working in collaboration with Oxitec to implement a novel genetics-based control strategy targeted at *Ae. aegypti*. Here, genetically modified mosquitoes have been released to suppress invasive and insecticide resistant *Ae. aegypti* populations. Release of an irradiated mosquito release program is also currently underway in Lee and St. John’s counties. Success in that trial could pave the way for larger scale field trials and applications. If there is to be widespread implementation of these novel approaches, there is a clear need for mosquito control personnel to be informed and participate. 

Our data highlight the fact that there are many understudied mosquito species where limited insight into the species, its interactions with other vectors, hosts and its preferred habitats could prove to be roadblocks for implementing both current and novel forms of control. This is an important knowledge gap to address in future research, and, ironically, one that could be overcome by applying other new tools and innovations to the problem. For instance, innovations such as artificial intelligence or machine learning could lead to the development of more rapid and effective mosquito surveillance tools and may facilitate the surveillance efforts across larger spatial and longitudinal settings. 

### 3.10. Study Caveats and Future Directions

The survey used in this study asked five questions to FMCDs, and these questions were limited in scope so that the responses did not provide an exhaustive view of all aspects of FMCD operation. For instance, certain aspects, such as how budget constraints might affect FMCD operations, were not asked. Independent taxing districts or country programs with varying degrees of resources likely influence control and surveillance capacity as well as responses to surveys from outside groups. Some of these aspects were already covered in a recent survey study [77]. Other aspects, such as the role of FMCDs in public outreach or education may benefit from future qualitative or quantitative evaluation to measure impact. It is expected that the current study will inform future surveys and follow-up questions that may help illuminate additional factors that influence mosquito surveillance and control efforts, and the role that research can play in supporting those efforts. 

## 4. Materials and Methods

Thirty-four out of sixty-six Florida Mosquito Control Districts responded to written surveys electronically. Questions included (1) their priority species for control and when these species are most active, (2) most common form(s) of adult mosquito control, (3) most common form(s) of larval control, and (4) research questions they would like to see addressed. The exact survey form used for data collection is provided in Supplementary S1. We gave 4 weeks to respond to our questions. Additional contacts were made via email or phone calls if we did not receive responses. The survey was closed after 8 weeks from initial contact. Once responses were collected, the data were organized in spreadsheets and tallied using Microsoft Excel. Plots were generated using Jupyter [78] and Python 3 [79] using Matplotlib [80], NumPy [81], and Pandas [82] library. Maps were generated using QGIS version 3.16 [83].

## 5. Conclusions and Recommendations

We have provided a snapshot of the current mosquito control priorities and practices for FMCDs. These data will serve as an important record of reference for comparison of mosquito control priorities and activities across regions. Here, we have provided important data on the priority species for control, particularly those that are largely understudied compared to well-known disease vectors such as *Ae. aegypti.* We believe it is likely that other regions, particularly those with a similar ecological setting and diversity of mosquito species to Florida, might face similar issues of understudied species as control priorities. Comparing priorities and approaches between Florida and these other regions may illuminate differences in mosquito control decision making, and also yield insight into intervention efficacy, and behavioral, phenotypic, or genetic differences of mosquito populations between regions.

Ideally, each regional jurisdiction should possess detailed information on all mosquito species recorded in their area, including vector status, activity time, history of insecticide resistant populations, larval habitat, and seasonal distribution, as this would allow them to select the most appropriate and effective control strategy to target any species. However, this would require training and extensive resources to execute and may not be feasible for districts with limited resources. Academic research can play an important part in filling these knowledge gaps, and an important first step in this process is for researchers to improve their understanding of mosquito control districts’ needs and priorities.

## Data Availability

The raw data used for this study are provided as Table S1.

## Supplementary Materials

The following are available online at https://www.mdpi.com/article/10.3390/pathogens10080947/s1, Table S1: Research questions identified by Florida Mosquito Control Districts. Supplementary S1: Survey form.
