# Supplementary material for: Mosquito Control Priorities in Florida—Survey Results from Florida Mosquito Control Districts
Source: Pathogens. 2021 Jul 28;10(8):947. doi: 10.3390/pathogens10080947 (PMC8401384; doi:10.3390/pathogens10080947)
Supplement: Supplementary file 1 [file pathogens-10-00947-s001.zip › Supplementary S1 - survey form.pdf]

Mosquito Control Priorities

...

Hi Yoosook, when you submit this form, the owner will be able to see your name and email address.

1. Provide the county name where your district is located

Enter your answer

2. Please name up to 3 priority species for mosquito control in your district.

Enter your answer

3. Please list two most common practices for adult mosquito control in your district.

Enter your answer

4. Please list two most common practices for larval control in your district.

Enter your answer

5. Please list up to two research questions you would like research community to address that will help your mosquito control efforts.

Enter your answer

Submit

This content is created by the owner of the form. The data you submit will be sent to the form owner. Microsoft is not responsible for the privacy or security practices of its customers, including those of this form owner. Never give out your password.
